# Laser assisted zona hatching does not improve live birth rate in patients undergoing their first ICSI cycles 

**Published:** 2013-12

**Authors:** Mohammad Hossein Razi, Iman Halvaei, Yasamin Razi

**Affiliations:** *Research and Clinical Center for Infertility, Shahid Sadoughi University of Medical Sciences, Yazd, Iran.*

**Keywords:** *Laser assisted hatching*, *Delivery rate*, *Clinical pregnancy*, *ICSI*

## Abstract

**Background:** Routine use of assisted hatching (AH) following ICSI is a controversial issue in the literature. There are rare studies regarding the effect of laser assisted hatching (LAH) on live birth rate.

**Objective: **Our main goal was to evaluate the effect of LAH on delivery rate as well as congenital anomaly in patients undergoing their first ICSI cycle.

**Materials and Methods:** A total of 182 patients subjected to ICSI were randomly aliquot into two groups of experiment and control. In experiment group, the embryos were subjected to LAH to open a hole in ZP (about 10-12 µm) while in control group, the transferred embryos were intact with no AH. The patients were followed for clinical pregnancy and delivery rate as well as congenital anomaly. All the patients were infertile due to male factor infertility and LAH and embryo transfer were done on day 2.

**Results:** Laboratory and clinical characteristics of two groups of experiment and control were the same. There were insignificant differences between two groups of experiment and control for clinical pregnancy rate (20% vs. 23.9%, respectively, p=0.3) and live birth rate (11.11% vs. 8.6%, respectively, p=0.6). Also no significant differences were observed between two groups of experiment and control for multiple pregnancy as well as congenital anomaly.

**Conclusion:** Routine use of LAH in first ICSI cycle for male factor patients may have no beneficial effects on clinical pregnancy and live birth rate.

## Introduction

Zona pellucida (ZP) hatching is natural process which is occurred after expansion of blastocyst and allows the embryo to implant into the uterine cavity. The blastocyst escapes from ZP with two probable mechanisms: ZP lysis by maternal or embryo (trophoectoderm) proteases and internal pressure from expanded blastocyst. Despite numerous achievements in assisted reproductive technology (ART), implantation rate has remained low and one of the causes of implantation failure could be failure in normal ZP hatching process (   1 , 2). 

Assisted hatching (AH), which was introduced more than two decades ago, showed the potential to increase the chance of implantation (3). First pregnancy following AH reported in 1988 and studies regarding the impact of AH have been followed till now. Several techniques have been introduced for embryo zona hatching (   4 , 5). Laser assisted hatching (LAH), which was proposed in early 90s, appears to be more safe compared to other AH techniques (5, 6). There are some indications for AH such as increased maternal age (≥40 years), increased FSH level, thick ZP (≥15µm), previous IVF failure (≥2), and frozen-thawed embryos (5,    7 -      10). Zona hardening which is due to in vitro culture or after freeze-thaw cycle and lack of produced proteases by embryo due to suboptimal culture condition are another indications of AH (11). Some investigators tried to assess the impact of AH based on etiology of infertility. Ciray *et al *reported the effect of AH on women with endometriosis (12).

Another controversial issue is AH performing for unselected patients. Antinori *et al* showed that AH may have positive effect on cases undergoing first IVF cycle while Tucker *et al* did not suggest use of AH for unselected patients (   13 , 14). Another study conducted by Hurst *et al*, showed that AH has no beneficial impact for good prognosis patients (15). Routine use of AH following ICSI is a matter of debate in the literature. Some believe that routine application of AH could increase pregnancy rate while others do not suggest AH as a general application for all embryos derived from ICSI procedure but none of them evaluated the effect of AH on live birth rate (14, 16). 

According to recent systematic review and meta-analysis, it is necessary to evaluate the effect of AH on live birth rate as well as congenital anomaly which can elucidate better conclusion in terms of efficacy and safety of clinical using of AH (17). To the best of our knowledge, there are rare studies regarding the effect of LAH on ICSI cases with male infertility that follow the outcome until live birth. Our main goal was to evaluate the effect of LAH on live birth rate as well as congenital anomaly in patients undergoing their first ICSI cycle. 

## Materials and methods

**Patient selection**

This randomized prospective study involved 182 infertile couples undergoing ICSI due to male factor infertility which were referred to our center from March 2009 to April 2010. Female factor infertility, egg donation cases, surrogacy, in vitro maturation cycles, conventional IVF cycles, frozen-thawed spermatozoa, frozen-thawed embryos, non-ejaculated spermatozoa, history of recurrent abortion or stillbirth as well as IVF failure were excluded. Only fresh ICSI was included in this study. Patients were randomized into two groups of experiment and control by computer generated random numbers. All the participants were signed the consent form. Also this study was approved by our center’s ethic committee.

**Controled ovarian hyper stimulation, oocyte recovery, ICSI, embryo evaluation and embryo transfer**

Ovarian hyper stimulation was done with 0.5 mg subcutaneously (S.C.) buserelin (super fact, Aventis, Germany) every day from day of 21 from menstrual cycle, then the dose of buserelin was reduced to 0.25 mg and ovarian stimulation would commence with 150-250 IU recombinant FSH (Gonal F, Sereno, Switzerland) S.C. Human chorionic gonadotropin (hCG) 10,000 IU (Pregnyl, Organon, Netherlands) was injected for egg retrieval schedule. Also ovarian responses during the artificial stimulation process were monitored with serum estradiol level and transvaginal sonography. 

Oocytes retrieval was performed 34-36 h after hCG injection under ultrasound guide. ICSI was performed according to the standard protocols which were previously described (18). Then the injected oocytes were washed several times and cultured in G1^TM^ V5 microdrop (Vitrolife, Gothenburg, Sweden) at 37^o^C incubator and 6% CO_2_ and high humidity (97%). All injected oocytes were evaluated for fertilization after 16-20 h. Embryo evaluation was done according to Hill *et al* criteria (19). Briefly, Grade A was considered as equal size blastomeres without any fragmentation. 

Grade B had slightly unequal blastomere up to 10% fragmentation. Grade C had unequal sized blastomeres up to 50% fragmentation with large granules. Grade D was considered unequal blastomeres with severe cytoplasmic fragments and large black granules. Grade A&B embryos were considered as high quality embryos. The grade D embryos were not transferred. Catheter used for embryo transfer (CCD, Laboratories C.C.D., France), embryo catheter loading technique as well as clinician who did embryo transfer were the same in both groups. Also one embryologist did embryo grading and LAH. 

Embryo transfer was done on day 2. Clinical pregnancy was determined by gestational sac visualization with aid of ultrasonography or by fetal heart beat detection after four weeks. Luteal phase support was continued until 12 weeks of gestation in case of positive pregnancy. The individual who followed the patients until delivery was blinded to groups.

**Laser Assisted Hatching**

In experiment group, in the morning of day 2, the embryos were subjected to LAH by Nikon TE300 inverted microscope (Nikon, Tokyo, Japan) which was equipped with Saturn system (Research Instruments LTD, UK). 1480 nm wave length infrared diode laser was used for 605 micro second duration to open a hole in ZP (about 10-12 µm) without any touching handles, also the operation was traced with a video monitor. After AH, the embryos were washed several time and left until embryo transfer time. In control group, the transferred embryos were intact with no AH.

**Statistical analysis**

Data was reported as mean±SEM. statistical analysis was performed using SPSS version 16 (Chicago, IL, USA). Independent samples t-test and chi-square or Fisher`s exact tests were applied for statistical analysis in quantitative and qualitative data, respectively. Also odds ratio with 95% confidence interval was reported for comparison of proportions. The odds ratios were referred to, high quality embryos, clinical pregnancy, live birth rate, multiple pregnancy and congenital anomaly. All hypotheses were two tailed and significant level was set at p-value less than 0.05.

## Results

Of 1318 retrieved oocytes, 975 were metaphase ІІ which were injected and formed 669 zygotes. Also 427 embryos were transferred in both groups. Mean female age, number of retrieved and metaphase ІІ oocytes and fertilized oocytes, number of formed high quality embryos as well as mean number of transferred embryos were similar in two groups (p>0.05, Table I). 

Of 182 couples which were followed, 18 and 22 cycles reached clinical pregnancy in groups of experiment and control, respectively. In experiment group, 10 babies were born and 8 cases reached delivery in controls. No significant differences for clinical pregnancy were found between two groups of experiment and control (20% vs. 23.9%, respectively, Table I). 

Live birth rate showed an increasing trend in experiment group compared to controls (11.11% vs. 8.6%, respectively), but the difference was insignificant (Table I). Two cases of multiple pregnancies (twin) were observed in each group. Only one congenital anomaly (kidney agenesis) was seen in experiment group.

**Table I:** Laboratory and clinical characteristics of cases in the experiment and control groups

**Variables**	**Experiment group** **(n=90)**	**Control group** **(n=92)**	**p-value**	**Odds ratio (95% CI)**
Age (Yrs) *	32. 9 ± 0.5	31.6 ± 0.4	0.06	
Number of retrieved oocytes*	6.72 ± 0.3	7.75 ± 0.4	0.09	
MІІ oocyte/cycle*	4.99 ± 0.2	5.7 ± 0.3	0.1	
Fertilized oocyte/cycle*	3.37 ± 0.2	3.98 ± 0.2	0.08	
2PN %*	70.17 ± 2.5	71.78 ± 2.5	0.6	
Number of transferred embryos*	2.42 ± 0.09	2.27 ± 0.08	0.2	
High quality embryos/cycle %	87.7	83.6	0.5	1.3 (0.6-3.2)
Clinical pregnancy rate/cycle (%)	18/90 (20)	22/92 (23.9)	0.3	0.7 (0.3-1.6)
Live birth rate (%)	10/90 (11.11)	8/92 (8.6)	0.6	1.3 (0.4-3.6)
Multiple pregnancy	2/10 (twins)	2/8 (twins)	1	0.6 (0.08-6.9)
Congenital anomaly	1/10	0/8	1	0.9 (0.7-1.1)

Independent samples t-test and chi-square or Fisher’s exact tests were applied for statistical analysis in quantitative (Age, Number of retrieved oocytes, MІІ oocyte, Fertilized oocyte, 2PN rate and Number of transferred embryos) and qualitative data (High quality embryos, Clinical pregnancy rate, Live birth rate, Multiple pregnancy and Congenital anomaly)

CI: confidence interval

MІІ: metaphase ІІ

PN: pronuclear

* : mean±SE.

## Discussion

Theoretically, AH can help better escape of embryo from ZP and some investigations have reported the positive effect of AH in poor prognosis women but also there are some reports that have shown AH does not improve rates of implantation and delivery in poor prognosis patients such as advanced maternal age and elevated FSH (   20 , 21). The other indications of AH have also the same story. Some investigators believe that AH can improve success rate in freeze-thawed embryos but the others did not prove it (   22 , 23). Also some researchers believe that thick ZP is not a suitable indication for AH (16). One probable causes of these discrepancies would be related to study power, study design or variation in AH technique used which is proposed by some authors (24). Because of heterogeneity between study results it seems AH outcome depends on patient’s characteristics. In current study we evaluated the effect of LAH on live birth rate in unselected patients undergoing their first ICSI cycle and the data showed that rate of live birth as well as clinical pregnancy rate, congenital anomaly and risk of multiple pregnancies were not increased. LAH was done in this study by one embryologist and the effect of operator would be omitted accordingly. 

We used laser for AH and according to Balaban *et al* there is no significant difference for outcome between different techniques of AH (25). So, the method used in current study may have little effect on results and the outcome is related to AH itself. We tried to omit confounding factor and the study groups were matched for laboratory and clinical characteristics. Also all of the patients had the same infertility etiology (male factor).

There are some studies regarding the effect of AH on good prognosis patients. There is also no general agreement to perform AH for unselected patients. It was shown that patients undergoing first IVF cycle may benefit from AH while others have shown implantation rate will not differ significantly in unselected patients (   13 , 14). Also Hurst *et al* designed a prospective pilot study on twenty good prognosis patients (13 AH, 7 control) (15). Their inclusion criteria were age ≤30 years, FSH ≤10 IU/l with normal semen and endometrial cavity or age ≤35 years with fertilization rate >50%. They reported no significant improvement in implantation and pregnancy rates following AH. Although their sample size seems to be not enough for final conclusion but our data support this hypothesis that AH does not improve the outcome with enough sample size and study power and also considering the final ART outcome: live birth. 

To our knowledge, it is first report about the effect of LAH on live birth rate and congenital anomaly in patients undergoing their first ICSI cycle. Our findings were similar to Sagoskin *et al* which were found no any effect of laser zona drilling on live birth in good prognosis patients (age <39 years, baseline FSH <10 mIU/mL, baseline E_2_ <75 pg/mL, first or second IVF cycle, good quality embryos) (26). Tucker *et al* demonstrated that AH will not improve pregnancy rate in ICSI cases and concluded that general application of AH could not improve ICSI outcome (14). Whereas Ali *et al* observed significant increase in clinical pregnancy rate using LAH in patient aged ≤36 years ,undergoing ICSI, when compared to ≥37 years and proposed routine use of AH in younger patients (16). But our data showed that routine application of LAH in ICSI cases does not increase live birth rate. Regarding the impact of AH on live birth rate, there are few trials in the literature and as it was shown in recent reviews, AH has no effect on live birth rate (17, 27). 

AH might lead to implantation of abnormal embryos. Although these abnormal embryos may abort during period of pregnancy but theoretically risk of abnormal born babies following AH should be considered. Follow-up of children born following use of diode laser showed that the risk of chromosomal abnormality and congenital malformation were not increased (21, 28). We only found one case with anomaly that was in experiment group (Table I). It seems the number be too small for statistically meaningful. The rate of fetal anomaly in present study was higher when compared to others and were in consistent with others (29-31). One of the limitations of this study would be lack of miscarriage rate report. Also it is suggested to long term follow-up of babies born from AH in order to elucidate probable long term effect of AH. 

One of disadvantages of using AH could be multiple pregnancy (32). Multiple pregnancies (monozygotic twin) were seen in both groups. Although more cases are required for better conclusion, but the results were in contrast with Hagemann *at al* (29). They reported monozygotic twin only in hatched group and also no significant differences were reported, as we did. Our results were also in consistent with Balakier *et al* (24). Although they used zona thinning technique for women aged <37 years with day 3 FSH baseline ≤10 IU/l, and ≤1 previous unsuccessful cycles and no significant differences were seen for multiple pregnancy. 

Martins *et al* also in recent meta-analysis concluded that AH will not increase the risk of multiple pregnancy in fresh embryo transferred to non-poor prognosis patients (17). Although it should be kept in mind that theoretically AH can improve the chance of multiple pregnancies so, the number of transferred embryos should be reduced in these cases. 

## Conclusion

LAH may not have any improvement on live birth rate in first ICSI cycle. So it seems application of this technique is not suggested in unselected cases. Regarding the potential risks of using LAH, it is recommended to select the patients with more scrupulosity. This study does not support routine use of LAH in ICSI cases.
